# The Syndrome of Absence Status Epilepsy: Review of the Literature

**DOI:** 10.1155/2014/624309

**Published:** 2014-02-10

**Authors:** Leonilda Bilo, Sabina Pappatà, Roberto De Simone, Roberta Meo

**Affiliations:** ^1^Epilepsy Center, Department of Neuroscience, School of Medicine, Federico II University of Naples, 80131 Naples, Italy; ^2^Institute of Biostructure and Bioimaging, CNR and Department of Biomorphological Sciences, Federico II University, 80131 Naples, Italy; ^3^Neurology Outpatients Service, Naples Local Health Unit, 80100 Naples, Italy

## Abstract

The authors review the literature for cases fulfilling the criteria for the proposed idiopathic generalized epilepsy syndrome (IGE) of absence status epilepsy described by Genton et al. (2008). Difficulties arising in diagnosing such cases are remarked, and possible overlapping with other proposed IGE syndromes is discussed.

## 1. Introduction

Absence status epilepticus (AS) is a peculiar epileptic condition which has been defined as a prolonged, generalized absence seizure, lasting at least more than half an hour but usually lasting for hours and even for days [1]; the impairment of consciousness is sometimes associated with automatisms or other subtle myoclonic, tonic, atonic, or autonomic phenomena [2]. AS has been distinguished in “typical,” if occurring in the setting of idiopathic generalized epilepsy (IGE) and “atypical” in patients with symptomatic or cryptogenetic generalized epilepsy [3]. During “typical” AS continuous or almost continuous generalized spike wave (SW) or polyspike-wave (PSW) activity at 2–4 Hz is recorded on EEG.

Typical AS may occur sporadically in many IGE syndromes, but it can also occur in a recurrent fashion as the main seizure type. This concept was underlined by Andermann and Robb in 1972 [4], commenting on the findings observed in a series of 38 patients with AS; the authors underlined that this condition could occur as the only seizure pattern or represent the dominant seizure pattern, especially in adult life. Most of the patients with recurring AS observed by these authors were either in the second decade of their life or adults, who had had or still had absence attacks. In adolescents AS was often sporadic, while in adults it had a greater tendency to recur. Generalized tonic clonic seizures (GTCS) terminating (or, more rarely, preceding) an episode of AS were also reported in this series.

Thirty-six years later, Genton et al. [5] described a series of 11 patients in whom recurrent, unprovoked episodes of typical AS represented the main clinical feature. The patients described in this series presented recurrent AS, with onset in adolescence or adulthood, as the main seizure type. Infrequent GTCS, mostly associated with AS, occurred in the majority of patients, while infrequent typical absences were reported in the minority of cases. While all patients had clinical and laboratory findings consistent with a diagnosis of IGE (normal neuroimaging, interictal EEG showing generalized SW, and PSW 2–4 Hz on a normal background activity), none of them could be included in any of the recognized IGE syndromes with typical absences or GTCS. The authors suggested that this condition may represent a specific and rare epilepsy entity which they proposed to name “absence status epilepsy” (ASE).

Reviewing the literature for eligible cases of ASE is not an easy task. While this may be partly due to the fact that this syndrome is rare, it must also be underlined that reports describing patients with typical AS in adult age do not necessarily give detailed information on recurrence of AS and its predominance among other seizure types. Moreover, especially in earlier reports, it is not always possible to ascertain whether or not a definite recognized IGE syndrome has been diagnosed in patients who presented with recurring AS. Consequently, while in some cases it is possible to evaluate if reported patients fulfill the criteria proposed by Genton et al. [5], in others a definite conclusion can not be reached due to lack of necessary data.

## 2. Review of the Literature

In reviewing the literature, patients were considered as eligible for a diagnosis of ASE if they satisfied the following criteria: recurrent episodes of unprovoked AS, onset of AS in adolescence or adult age, AS being the main seizure type, and clinical and laboratory findings consistent with a diagnosis of IGE but not suggesting one of the recognized IGE syndromes. We considered that definite cases of ASE are those who fulfilled all the above mentioned criteria and possible cases are those in whom some features were atypical or in whom data were insufficient for a diagnosis of ASE. The main data of reviewed patients are summarized in Table 1.

We performed a literature search using the following keywords, alone or in combination: absence status epilepsy, absence status, nonconvulsive status, recurring/recurrent absence status, recurring/recurrent nonconvulsive status, absence status in adults, and nonconvulsive status in adults. The literature search was performed on PubMed, PubMed Central, and Google Scholar.

On the whole, 106 articles were retrieved. We selected for this review only reports describing recurrent AS with onset in adolescence/adulthood, in which individual patients' data (regarding in particular age at onset of AS, recurrence of AS, characteristics of coexisting seizures, and diagnosis of epilepsy syndrome) were detailed or could be inferred, albeit incompletely in some cases.

Patients eligible for a diagnosis of ASE are more easily found in single case reports, probably because these case presentations include a detailed clinical history in which all the data necessary for a diagnosis are given. This is the case of the patients reported by Nightingale and Welch [6], Zambrelli et al. [7], Bilo et al. [8], and Pro et al. [9], who all fulfill the criteria suggested for diagnosis of ASE.

Nightingale and Welch [6] describe a female patient in whom recurring AS was diagnosed at 66 years. In childhood she had suffered from GTCS, which had recurred after a long remission at 47 years; after this relapse, she had been treated with primidone and phenytoin. No overt epileptic seizures had appeared since then, but at 56 years she had started to present long and recurring episodes of “vagueness,” which recurred quite regularly at 4-week intervals and lasted about 48 hours. At the age of 66 a diagnosis of AS was made after recording of ictal EEG showing almost continuous generalized SW activity at 2-3 Hz. Neurological examination was normal; no information on family history is offered. Interictal EEG showed bursts of generalized SW; photosensitivity is not mentioned. The patient refused to increase/modify her previous antiepileptic treatment and continued to present AS episodes.

Zambrelli et al. [7] report a male patient without family history of epilepsy who presented a GTCS at the age of 20. Successively he presented recurring AS, generally followed by GTCS, which were initially sporadic but became very frequent after the age of 73. No absences are reported. AS lasted from 48 to 72 hours, with ictal patterns characterized by continuous generalized SW at 3 Hz. Interictal EEG showed brief 3-Hz generalized SWD, without photosensitivity. Several AEDs, alone or in association, failed to control the AS episodes; at the time of the report, seizure control had been obtained for 2 years after adding lamotrigine (LTG) to valproate (VPA).

Bilo et al. [8] report a 56-year old male who had been suffering from episodes of clouded consciousness and confusion of long duration (36–48 hours) since adolescence. The episodes were initially rare, but during adulthood their frequency increased progressively up to about 1/month; moreover, he presented 2 GTCS which prompted him to seek medical attention. He had no family history of epilepsy. Interictal EEG showed frequent bursts of generalized SW and PSW, without photosensitivity; ictal EEG showed almost continuous generalized SW or PSW activity at 2-3 Hz. He underwent a [18F]FDG-PET study, which showed relative hypermetabolism in thalami bilaterally, mostly in the anterior nuclei, and relative hypometabolism in bilateral frontal cortex and, to a lesser extent, relative hypermetabolism in the cerebellar vermis and relative hypometabolism in parietal and posterior cingulate cortices and in cerebellar hemispheres. Interictal [18F]FDG-PET was normal. LTG monotherapy was uneffective, and side effects prevented the use of adequate doses of VPA; treatment with LTG and low doses of VPA led to reduction, but not disappearance, of AS episodes [10].

Pro et al. [9] report a female patient who presented recurring episodes of mental confusion and ideomotor slowing since the age of 54, usually lasting 5-6 h, with a frequency of 2/3 per year. Medical observation was sought at 77 years. No other seizures had ever occurred, and no triggering factors for the AS were observed. Interictal EEG showed 3–3.5 Hz generalized SW and PSW and photoparoxysmal response; ictal EEG showed continuous, diffuse 3-4 Hz SW and PSW. LTG treatment controlled the AS episodes. A 16-year-old grandson had had febrile seizures and an episode of loss of consciousness that was not clearly interpreted as epilepsy; his EEG recordings showed epileptiform activity which was focal during sleep and generalized during wakefulness.

Other individual case reports found in the literature describe recurring AS in which, however, some atypical features leave doubts as to their eligibility for a diagnosis of the ASE syndrome as described by Genton et al. [5]. This is the case for the patients described by Terzano et al. [11], Iivanainen et al. [18], and Fernández-Torre and Rebollo [19].

Terzano et al. [11] report a woman with no family history of epilepsy and a normal neurological history until the age of 70. At the age of 70 she presented a GTCS and started treatment with barbiturates and phenytoin. Six years later she began to present recurring confusional episodes which lasted as long as 24 hours, often terminating with GTCS, which led to medical observation. The patient was observed during two distinct episodes; in both she was slowed and confused and presented massive and diffuse myoclonic jerks involving mainly the abdominal muscles and the antigravitative muscles of the upper limbs. Ictal EEG showed slowed background activity with superimposition of generalized polyspikes and PSW, grouped in continuous and arrhythmical bursts; an increase in the paroxysmal discharges and the myoclonus was produced by intermittent photic stimulation. Interictal EEG showed isolated diffuse PSW and SW. The history of this patient is in agreement with a possible diagnosis of ASE: onset in late adulthood, recurrent AS as the main seizure type, and concomitant GTCS. However, the clinical and EEG features of her status episodes are quite unusual and actually, rather than AS, can be considered as myoclonic absence status epilepticus as defined by the ILAE task force report [23]. However, while AS with prominent myoclonic features is usually seen in IGE patients who present seizures with myoclonic jerks, this patient only presented GTCS aside from episodes of status. It is possible that in the future the definition of ASE could include also patients with recurring myoclonic absence status, if they share the other features which characterize this condition (recurring absence status as the main seizure type and lack of inclusion in any recognized IGE syndrome). To date, however, the peculiar features of status do not consent to include confidently this patient into the present definition of ASE.

Iivanainen et al. [18] report 2 female patients with recurring AS with onset in adulthood. In both cases, however, AS does not seem as the prominent seizure type. Patient 1 had an undefined IGE with onset of GTCS in childhood, absence seizures appearing at 32, and recurring AS at 55. All seizure types were quite frequent and concomitant seizures persisted at the time of AS onset. Patient 2, conversely, had epilepsy onset at 57 years, with sporadic absences (which successively increased in frequency) and a single GTCS followed from a confusional state, possibly resulting in AS, at the age of 61. No family history for epilepsy is mentioned. A definite AS occurred a couple of weeks later, with hospitalization and recording of continuous generalized SW activity. No photosensitivity was present in ictal and interictal EEGs. She was promptly treated with VPA and became completely seizure free. While the history of this patient closely resembles those of patients in the series of Genton et al. [5] (onset in later life of undetermined IGE, recurring AS, associated sporadic GTCS, and absences), recurrence of AS is too low for these seizures to be considered the main seizure type with regard to absences and GTCS. However, one may argue that the prompt diagnosis and treatment of AS (which occurred after the second episode) may have altered the natural course of the condition in this patient. In fact, in most patients with recurring AS, the diagnosis of status occurs quite late and several episodes occur before an appropriate treatment approach is employed. Similar considerations may be suggested for the case described by Fernández-Torre and Rebollo [19], who report an elderly woman who at the age of 68 presented with an episode of typical AS as late complication of an unrecognised and “nonspecific” picture of IGE. Ictal EEG showed frequent and recurrent generalized paroxysms of 4–6 Hz PSW and SW intermixed with brief periods of normal background activity, while the interictal EEG showed occasional discharges of generalized SW and a photoparoxysmal response. The patient was treated with VPA and remained seizure free during the 2-year follow-up. We have no evidence that this patient could have shown a tendency to AS recurrency and that AS may have resulted in being her main seizure type. However, the possible role of the well documented prompt diagnosis and treatment in influencing the natural history of her epilepsy must be taken into account. Accordingly, the authors remark the similarities between their patient and the one described by Genton et al. [5].

Other individual reports (Szucs et al., 2008; Mireles and O'Donovan, 2010) do not give sufficient data for a definite diagnosis of ASE. Szucs et al. [16] present 3 female patients with AS with onset in adult age. None of them seems to fit into a possible diagnosis of ASE, even if additional information/clarification on their clinical histories would be needed before reaching a definite conclusion. Patient 1 has a diagnosis of juvenile absence epilepsy (JAE), but the onset of her absence seizures is reported at 6 years of age. She suffered from absences and GTCS, whose frequency is not detailed but seems to be quite high. Only one AS was documented at the age of 55. Also patient 3 had a single AS episode occurring at 63 after a convulsive status; she suffered from epilepsy since the age of 15, manifested as frequent GTCS which the authors consider as probable episodes of ASE, without offering any explanation for this diagnosis. Only patient 2, with a diagnosis of undefined IGE with onset around 45 years of age and recurring long confusional states (recognized as AS at the age of 55 after an ictal recording), could fit into the diagnosis of ASE. However, this patient also presented GTCS probably with high frequency, and the authors do not clarify if AS were the prominent seizure type.

In a report presented as a poster, Mireles and O'Donovan [17] report 3 patients with recurrent AS with onset in adult life and presumed IGE. The limited amount of data typical of a poster presentation does not consent a detailed clinical history; patients' sex is not detailed, but a male preponderance is mentioned in the discussion. Patient 1 had had absences in childhood, responsive to ethosuximide; no additional details are given, so a possible diagnosis of childhood absence epilepsy (CAE) can not be ruled out. At the age of 49 the patient presented recurrent confusional episodes, diagnosed as AS, which failed to respond to several antiepileptic drugs and were finally controlled by levetiracetam. Patient 2 had adult onset (60 years of age) of yearly bouts of AS evolving to GTC status; while convulsive status was controlled, AS was drug resistant. Patients 3 had epilepsy onset at 47, with recurring unusual electrographic generalized status, lasting for days, during which complex neuropsychological testing was performed without difficulty. While apparently in all patients, ictal EEGs consistent with a diagnosis of AS was obtained, the authors underline “lack of correlation between epileptiform patterns on EEG and precise manifestations of altered awareness and seizure frequency rates.” Family history and response to intermitted photic stimulation are not reported in any of the patients. To our knowledge these authors have not reported these cases outside the poster presentation. Without additional information on these interesting patients, it is not possible to understand if they might actually represent cases of ASE.

Many authors have reported patient series with recurrent adult onset AS. In most of these reports, no patient is eligible for a diagnosis of ASE because all of them suffer from a definite recognized IGE syndrome and/or have isolated episodes of AS and/or AS is not the main seizure type in their clinical history (Michelucci et al., 1996 [20]; Baykan et al., 2002 [21]; Nguyen Michel et al., 2011 [22]).

In other reports describing patient series, the lack of detailed individual data does not allow to ascertain if ASE could be diagnosed, at least in a minority of patients. Lee [12] describes 11 adult patients with AS without previous history of epileptic seizures, with a follow-up lasting as long as 5 years. Two of these patients are reported to have partial seizures during follow-up, casting some doubts on the previous diagnosis of AS. Moreover, in 7 patients of this series AS was provoked by metabolic imbalance or related to psychotropic treatment, suggesting that they may suffer from “de novo” AS, a condition reported in middle age or in the elderly, without previous history of epilepsy, resulting from the addition of several epileptogenic factors (iatrogenic or metabolic) [24]. Recurrence of status during follow-up is reported in 3 patients, after discontinuation of antiepileptic treatment. However, there is no way of ascertaining if patients with recurring AS had also other generalized (or even focal?) seizures as their main seizure type, if a recognized epilepsy syndrome was diagnosed and if they had had a situation-related provoked status to begin with.

Similarly, no definite conclusion can be drawn from the report by Tomson et al. (1992) [14], describing 32 patients with nonconvulsive status, in some of whom a review of case histories disclosed probable, previously undiagnosed episodes of status. Twenty-one of these patients presented recurrence of AS on follow-up. Eighteen patients had “generalized EEG seizure activity” during the status, but 2 of them were finally diagnosed as suffering from focal epilepsy. Of the remaining 16, 6 had a history of a recognized IGE syndrome, 5 had a diagnosis of undetermined epilepsy due to insufficient information of previous seizures, and 5 had had AS as the only epileptic manifestation. Since it is not possible to ascertain if some of these 5 patients were in the group of previous undiagnosed episodes of status or if they were in the group of patients with recurring status on follow-up, it is not possible to know if these patients had recurring AS and no conclusion can be reached as to the possible diagnosis of ASE.

Other reports describing patient series give more detailed information on individual cases, allowing the proposal of possible diagnosis of ASE in some cases. Berkovic et al. (1989) [13] report the effectiveness of VPA treatment in preventing recurrence of AS in 25 patients (this report includes also the patients with AS discussed, with no individual details, in the paper from Andermann and Robb mentioned above [4]). The series from Berkovic et al. [13] includes 18 patients with IGE, in 16 of whom AS was recurring; in 14 of these 16, the onset of AS was after 15 years of age. In the table no information is given about the specific IGE syndrome presented by individual patients, and it is not specified if AS was the main seizure type in any of these patients. However, in the discussion the authors underline that “the group [with IGE] included patients with typical IGE [⋯] but other patients were of the unusual but well recognized group of IGEs beginning in middle to later life where AS is a particular prominent seizure type.” This definition is more or less identical to the definition of ASE given by Genton et al. [5], and so we must infer that this series most probably includes patients with ASE. It is not possible, however, to gather how many of these patients are presented nor their individual features such as sex, age at onset of AS, family history, and photosensitivity.

In 1998 Agathonikou et al. [15] reported a series of 21 adult IGE patients who had presented one or more episodes of typical AS, with the aim of documenting the relations of AS to the various syndromes of IGEs. The original study population consisted of 136 adult patients with IGE, of whom 21 had presented AS. All these 21 patients had presented typical absences and GTCS besides AS; in most patients AS had recurred, with varying frequency (from 2/life to more than 40/life); precipitating factors for AS were occasionally reported, but only in 3 patients a consistent precipitant was identified. Most patients could be diagnosed with a specific IGE syndrome, with the highest frequency of AS observed in perioral myoclonia with absences (PMA) (57.1% of all PMA adult patients observed in the study population) followed by IGE with phantom absences (IGE-PA) (46,2%). However, half of the patients with PMA presented only one episode of AS in their life, while patients affected by IGE-PA had the highest mean of AS episodes during life. Interestingly, 4 of the 21 patients of this series could not be included in any definite IGE syndrome and were consequently defined as suffering from “unclassified IGE with typical absences.” There is no information on family history or photosensitivity in these 4 patients; in all of them AS had recurred, with episodes ranging from 4 in life to more than 30. Three patients (n° 18, 19, and 20) had onset of AS in adult age. All presented other seizure types consisting of absences and GTCS; it is not possible to ascertain if AS was the main seizure type in this group, but this possibility seems likely when AS recurs very frequently. In conclusion, patient number 18 seems a likely candidate for a diagnosis of ASE, with >30 episodes of unprovoked AS with onset at 48 years of age; in patients number 19 (4 episodes of AS) and in patient number 20 (7 episodes of AS) no definite conclusion can be drawn.

## 3. Conclusions

While the existence of a seizure condition with recurrent, unprovoked AS in adult age has been recognized 40 years ago, only recently this condition has been proposed as a possible definite entity in the IGE group [5]. The proposing authors [5], presenting a homogeneous group of 11 patients with this condition, define its features as recurrent AS with onset in adolescence or adulthood, infrequent GTCS, rarely typical absences, no family history of epilepsy, normal neuroimaging, interictal EEG showing generalized SW and PSW 2–4 Hz on a normal background activity, absence of photoparoxysmal responses, variable response of AS to IV BDZs, and good seizure control with adequate dose of antiabsence drugs, mainly VPA.

Recurrent unprovoked ASs are reported in another proposed adult IGE syndrome, IGE-PA, characterized by phantom absences (PA), infrequent GTCS, and AS in up to 50% of the patients [25]. PAs are absence seizures so mild and short-lasting to be barely perceived by the patient or the observer, with a duration of approximately 2–4 s without other clinical features [26]; they can only be revealed by cognitive tests during video-EEG monitoring. PAs are difficult to diagnose and often escape recognition not only by patients but by physicians as well. Consequently, while we have excluded from our review all reports in which a diagnosis of IGE-PA was proposed, it is not possible to rule out the occurrence of PA in the reports which we have suggested as ASE patients. Genton et al. [5], while underlining that patients in their series present some clinical features overlapping with patients with IGE-PA who also present recurrent AS, find episodes possibly consistent with PA in only one of their patients; they also remark, however, that it is not possible to exclude in their series subtle cognitive deficit during the generalized paroxysmal discharges lasting for more than 3 s. These authors [5] question the recognition of IGE-PA as a specific epilepsy condition: the same doubts, however, may be cast on the recognition of the ASE syndrome, which at present is not considered as a distinct IGE entity. Actually, at present it is probably questionable to consider any of these two conditions as specific IGE syndromes, and it is possible that both may share the same pathophysiologic mechanisms and anatomical substrates of more common phenotypic expressions of IGE. However, it is conceivable that the epileptic conditions characterized essentially by recurring AS as the main seizure type might in the future be considered as a specific IGE entity, with ASE and IGE-PA being viewed as different parts of the same continuum. More reports of patients with recurrent AS, with specific investigations aimed at disclosing PA, are warranted to clarify this point.

Until recently, AS occurring in adult age was often unrecognized and misdiagnosed as complex partial status epilepticus or, especially in the elderly, as a confusional episode related to cerebrovascular disturbances. Emergency EEG is of paramount importance for the diagnosis of AS. Moreover, AS occurring in elderly patients with IGE must also be distinguished from “de novo” AS, a condition first reported by Thomas et al. [24] and occurring in patients without previous history of epilepsy under the influence of several epileptogenic factors. Most commonly, it is caused by withdrawal of psychotropic drugs, usually benzodiazepines; metabolic imbalance or chronic alcoholism may act as cofactors. De novo AS is considered a situation-related epileptic status epilepticus; long term antiepileptic treatment is not required, since there is no recurrence if precipitating factors are avoided. However, as underlined by Fernández-Torre and Rebollo [19], the differential diagnosis between “de novo” AS and AS occurring in patients with IGE may be difficult, since elderly subjects may often overlook a previous history of epilepsy, and the use of psychotropic drugs in these patients is quite common. A detailed clinical history and analysis of interictal EEGs are necessary to avoid misdiagnosis, which may lead to mistreatment and, most often, to recurrence. However, in recent times a better recognition of this entity has led to prompt diagnosis and early appropriate treatment, with a consequent reduction/disappearance of recurrences, as described in some reports [18, 19]. In consideration also of the usual good response to treatment, it is conceivable that in the future one of the cardinal features of this proposed syndrome—recurrence of AS—might gradually disappear.

## Figures and Tables

**Table 1:** Patients with recurrent AS with onset in adolescent/adult age.

Authors	Sex	Age at AS onset (yrs)	Other seizures	Epilepsy family history	Photo sensitivity	AED therapy	Response of AS to AEDs	Notes
Definite cases of ASE								

Nightingale and Welch, 1982 [6]	F	56	GTCS	NS	NS	PRI, PHT	Persistence of seizures	

Zambrelli et al., 2006 [7]	M	>20	GTCS	No	No	LTG, VPA	Seizure free	

Genton et al., 2008 [5]	F	14	GTCS	No	No	VPA	Seizure free	
	F	34	A	No	No	VPA, LTG	Seizure free	
	F	16	GTCS	No	No	VPA, ESM, and CZP	Persistence of seizures	
	M	35	GTCS	No	No	VPA, ESM	Seizure free	
	M	15	GTCS	No	No	VPA	Seizure free	
	M	26	GTCS	No	No	VPA	Seizure free	
	F	26	GTCS	No	No	VPA, PB, and LEV	Persistence of seizures	
	F	65	GTCS	No	No	VPA	Seizure free	
	M	16	A, GTCS	No	No	VPA, LTG	Persistence of seizures	
	M	36	A	No	No	LEV, TPM	Seizure free	
	M	36	GTCS	No	No	LTG, ESM	Seizure free	

Bilo et al., 2010 [8]	M	14	GTCS	No	No	LTG, VPA	Persistence of seizures	

Pro et al., 2011 [9]	F	54	No	Yes	Yes	LTG	Seizure free	

Possible cases of ASE (atypical features)								

Terzano et al., 1978 [11]	F	76	GTCS	No	Yes	?	?	Atypical feature: prominent myoclonic jerks during AS

Possible cases of ASE (insufficient data)								

Lee 1985 [12]	Three patients in this series presented recurring AS, but it is not clear if a defined IGE syndrome and/or other seizures were the main seizure type and/or if AS episodes were situation related.							

Berkovic et al., 1989* [13]	Fourteen patients in this series had recurring AS with onset after the age of 15. An undetermined number of these 14 patients belonged to “the unusual but well-recognized group of IGEs beginning in middle to later life, where AS is a particular prominent seizure type.”							

Tomson et al., 1992 [14]	AS was the only seizure type in 5 patients with undetermined IGE. However, it is not clear whether or not AS was recurring in any of these patients.							

Agathonikou et al., 1998 [15] (patients 18, 19, and 20)	M	48	GTCS	NS	NS	VPA, CBZ, and PHT	Seizure free	It is not clear if AS is the main seizure type.
	F	36	A, GTCS	NS	NS	VPA	Seizure free	It is not clear if AS is the main seizure type.
	F	43	A, GTCS	NS	NS	PB, PHT, ESM, and CZP	Seizure free	It is not clear if AS is the main seizure type.

Szucs et al., 2008 [16] (patient 2)	F	45	GTCS	NS	NS	NS	Seizure free	It is not clear if AS is the main seizure type.

Mireles and O'Donovan, 2010 [17] (patients 1, 2, and 3)	NS	49	A	NS	NS	LEV	Seizure free	Possible diagnosis of CAE
	NS	60	GTCS	NS	NS	LEV, LTG, PTH, and VPA	Persistence of seizures	Insufficient clinical data
	NS	47	No	NS	NS	VPA	Seizure free	Insufficient clinical data

Possible cases of ASE (prompt diagnosis may have altered natural history)								

Iivanainen et al., 1984 [18] (patient 2)	F	61	A, GTCS	No	No	VPA	Seizure free	This patient had few seizures in her life, among which AS does not stand as the main seizure type. However, its low recurrence may be due to prompt diagnosis and treatment.

Fernández-Torre and Rebollo, 2009 [19]	F	68	GTCS	No	Yes	VPA	Seizure free	Concomitant seizures were rare and disappeared after childhood. AS is not recurrent, but this may be due to prompt diagnosis and treatment.

Cases of AS not fulfilling criteria for ASE								

Iivanainen et al., 1984 [18] (patient 1)	F	55	A, GTCS	No	No	VPA, PB		Concomitant seizures were very frequent.

Michelucci et al., 1996 [20] (patients 1 and 4)	F	28	A, GTCS	No	NS	VPA	NS	AS was not the main seizure type.
	M	41	A, GTCS	No	NS	PHT, PB	NS	Diagnosis of JAE.

Agathonikou et al., 1998 [15]	Patients 1–17 had defined IGE syndromes; moreover, patients 9, 10, 16, and 17 had presented only one AS episode.							

Baykan et al., 2002 [21]	All 8 patients had recurrent AS but all of them were diagnosed with defined IGE syndromes.							

Nguyen Michel et al., 2011 [22] (patient 5)	F	71	A, GTCS	NS	NS	LTG, LEV	?	Diagnosis of CAE.

AS: absence status; ASE: absence status epilepsy.

*This series includes also the patients presented in Andermann and Robb, 1972 [4].

GTCS: generalized tonic clonic seizures; A: absences.

NS: not specified.

AED: antiepileptic drugs; PRI: primidone; PHT: phenytoin; LTG: lamotrigine; VPA: valproate; ESM: ethosuximide; CZP: clonazepam; PB: phenobarbital; LEV: levetiracetam; TPM: topiramate; CBZ: carbamazepine.

IGE: idiopathic generalized epilepsy; JAE: juvenile absence epilepsy; CAE: childhood absence epilepsy.
